# A novel therapeutic anti-HBV antibody with increased binding to human FcRn improves *in vivo* PK in mice and monkeys

**DOI:** 10.1007/s13238-017-0438-y

**Published:** 2017-07-04

**Authors:** Ciming Kang, Lin Xia, Yuanzhi Chen, Tianying Zhang, Yiwen Wang, Bing Zhou, Min You, Quan Yuan, Chi-Meng Tzeng, Zhiqiang An, Wenxin Luo, Ningshao Xia

**Affiliations:** 1 State Key Laboratory of Molecular Vaccinology and Molecular Diagnostics, National Institute of Diagnostics and Vaccine Development in Infectious Diseases, School of Public Health, School of Life Science, Xiamen University, Xiamen, 361105 China; 2 Key Laboratory for Cancer T-Cell Theranostics and Clinical Translation (CTCTCT), Translational Medicine Research Center, School of Pharmaceutical Science, Xiamen University, Xiamen, 361105 China; 3 Texas Therapeutics Institute, The Brown Foundation Institute of Molecular Medicine, University of Texas Health Science Center at Houston, Houston, TX 77030 USA

**Dear Editor,**

Antibody immunotherapy is a well-established therapeutic modality for cancer, acute viral infections (Marasco and Sui, 2007) and persistent viral infection such as HIV (Barouch et al., 2013) and HCMV (Freed et al., 2013). To reduce immunogenicity of rodent antibodies (Abs), approved antibody drugs entering clinical trials are of human origin or are humanized versions of rodent antibodies (Reichert, 2008). Recently, there is a strong drive to improve therapeutic efficacy, reduce cost, and provide convenient dosing to patients by designing next-generation antibodies with improved pharmacokinetic properties and modulated immune effector functions (Grevys et al., 2015). The neonatal Fc receptor (FcRn) is a heterodimer that comprises transmembrane α chains and β_2_-microglubulin (β_2_m). Optimizing FcRn-IgG interaction through Fc engineering is an effective strategy to improve pharmacokinetic (PK) or pharmacodynamics (PD) properties of therapeutic antibodies (Datta-Mannan et al., 2007). Increased affinity of the FcRn-IgG interaction at pH 6.0 and/or 7.4 has resulted in improved terminal phase half-life (t_1/2_) of antibodies *in vivo* (Dall’Acqua et al., 2002).

In this study, five Fc variants known to enhance human FcRn (hFcRn) binding with mutations in the C_H_2 and/or C_H_3 domains were constructed on a humanized version of E6F6 (huE6F6), a novel therapeutic mAb against HBV. This mAb binds to an unique epitope on HBsAg and potently suppress levels of HBsAg and HBV DNA for several weeks in HBV transgenic mice (Zhang et al., 2016). All five Fc variants showed binding to hFcRn increased by a factor of up to 60-fold at pH 6.0 when compared to wild-type huE6F6 (WT huE6F6). A competitive binding assay was developed to identify the candidate suitable for further pharmacokinetic studies. Finally, huE6F6 Fc mutant M252Y/S254T/T256E (huE6F6-YTE) showed considerably longer serum half-life than the wild-type antibody in both mouse and cynomolgus monkey models. Taken together, these results provide a PK-improved immunotherapeutic agent, the first Fc-modified humanized antibody against chronic HBV infection (CHB).

To obtain huE6F6 IgG1 Fc variants with enhanced PK properties, several Fc-engineered variants were made by substitution of amino acid residues at the C_H_2-C_H_3 interface, which have been reported to modulate binding to hFcRn, transplacental transport, and serum half-life. Our preliminary results suggested that only these five Fc mutants, T307A/E380A/N434A (AAA) (Petkova et al., 2006; Yeung et al., 2010), M252Y/S254T/T256E (YTE) (Dall’Acqua et al., 2006; Zalevsky et al., 2010; Robbie et al., 2013), T250Q/M428L (QL) (Hinton et al., 2005), M428L/N434S (LS) (Zalevsky et al., 2010), and N434S (N/S) (Zalevsky et al., 2010) displayed increased level in hFcRn binding compared with WT huE6F6 (data not shown). HBsAg specific chemiluminescent enzyme immunoassay (CLEIA) of titrated Abs showed that these Fc mutants bound equally well to HBsAg, indicating that the Fc mutations had no effect on HBsAg binding (Fig. 1A).

**Figure 1:**
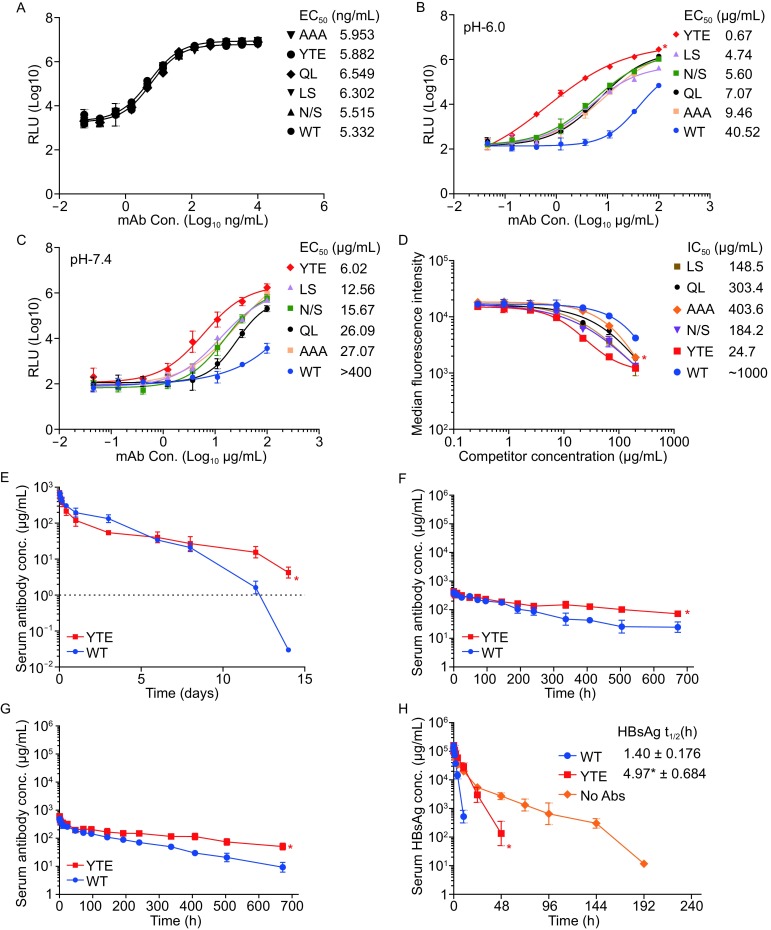
***In vitro*** **characterization and** ***in vivo*** **PK profile of WT huE6F6 and Fc-engineered variants**. (A) Binding of anti-HBV WT huE6F6 and Fc-engineered variants to HBsAg. WT, wild-type humanized E6F6 antibody; RLU, relative luminescent units; Con., mAb concentration. CLEIA binding of anti-HBV WT huE6F6 and Fc-engineered variants (100–0.045 μg/mL) to hFcRn at pH 6.0 (B) and pH 7.4 (C), respectively. Serial antibody dilutions were added to hFcRn-coated (1 μg/mL) plates in PBS (pH 6.0 or pH 7.4) and incubated for 1 h at room temperature, prior to washing using PBST (pH 6.0 or pH 7.4). The EC_50_ titers, representing the concentration for 50% of maximal binding, were calculated by GraphPad Prism. Data presented is mean ± SEM of one representative experiment out of three. (D) Flow cytometry-based competitive binding assay of Fc variants using hFcRn-transfected MDCK cells. Dylight-594 labeled human IgG was used as competitor. IC_50_ representing the concentration for 50% of inhibition, were calculated by GraphPad Prism. Data presented is mean ± SEM of one representative experiment out of three. *, data for the Fc mutant group is significantly different compared with WT huE6F6, with a *P* < 0.05 (Student’s *t*-test). (E) Serum antibody concentrations (μg/mL) of WT huE6F6 (blue) or YTE variant (red) following a single i.v. dosing of 10 mg/kg to hFcRn transgenic mice (*n* = 6 animals per antibody). (F) Serum antibody concentrations (μg/mL) of WT huE6F6 (blue) or YTE variant (red) following a single i.v. dosing of 20 mg/kg to cynomolgus monkeys (*n* = 3 animals per antibody). (G) Serum concentrations (μg/mL) for antibody treatment group of WT huE6F6 (blue) or YTE variant (red) following a single i.v. dosing of CHO-HBsAg at 3 mg/kg followed by antibody intravenous injection at 20 mg/kg to cynomolgus monkeys (*n* = 3 animals per antibody). A quantitative CLEIA was used to monitor the serum concentrations of the tested antibodies following HBsAg specific binding assay. (H) Pharmacokinetic profile of CHO-HBsAg for antibody treatment group in cynomolgus monkeys. Serum concentrations (μg/mL) of CHO-HBsAg following a single i.v. dosing of CHO-HBsAg at 3 mg/kg (orange), and then followed by huE6F6 WT treatment (blue) or YTE variant treatment (red) at 20 mg/kg to cynomolgus monkeys. Terminal half-life of CHO-HBsAg in cynomolgus monkeys was displayed above. t_1/2_, terminal half-life; CL, the volume of serum antibody cleared per unit of time; AUC_inf_, the area under the concentration-time curve extrapolated from time zero to infinity; i.v., intravenous; Conc., mAb concentration

As hFcRn binds human IgG (hIgG) at the lower pH of the early endosome (pH 6.0–6.5) and dissociates at the neutral pH of blood (pH 7.4), we established a CLEIA assay to screen antibodies for hFcRn binding at pH 6.0 and pH 7.4, respectively. As expected, hFcRn was shown to bind WT huE6F6 and Fc mutants in a strictly pH-dependent manner, with strong binding at pH 6.0 but reduced interaction at pH 7.4 (Fig. 1B). Furthermore, a side-by-side comparison of the five Fc-engineered variants revealed that they all bound more strongly to hFcRn than did wild-type (WT) by a factor of 4 to 60 at pH 6.0, with a hierarchy from strongest to weakest binding as follows: YTE > LS > N/S > QL > AAA > WT (Fig. 1B). Significant increased level was detected in YTE variant group as compared with WT huE6F6 group (Fig. 1B, *P* < 0.05). The binding of Fc variants at pH 7.4 were comparably improved with the same ranking of binding affinity as that at pH 6.0 (Fig. 1C).

To compare Fc-engineered antibodies and WT huE6F6 in a flow cytometry-based competitive assay of binding to hFcRn at pH 6.0, we constructed a human FcRn-transfected Madin-Darby canine kidney (MDCK) epithelial cell line. Dylight-594 labeled human IgG was used as competitor. Comparison of the IC_50_ values indicated that the YTE variant with IC_50_ values of 24.7 μg/mL performed about 40-fold better than did WT huE6F6 in competitive binding to hFcRn at pH 6.0 (Fig. 1D, *P* < 0.05), which was used in analyzing the PK behavior *in vivo*. Other Fc variants showed comparatively lower IC_50_ values of 100–500 μg/mL (Fig. 1D).

The PK properties of YTE Fc variant and WT huE6F6 were examined in hFcRn transgenic mice following a single intravenous (i.v.) dose of 10 mg/kg antibody (*n* = 6 animals per antibody). The relevant PK parameters and average serum concentration time profiles were shown in Table 1 and Fig. 1E, respectively. As expected, the YTE variant, with substantial binding to hFcRn at both acidic and neutral pH, showed terminal half-life significantly extended by 1.5-fold compared with WT huE6F6 in mice (WT, t_1/2_ = 20.1 ± 7.5 h; YTE, t_1/2_ = 30.9 ± 10.8 h; *P* < 0.05; Table 1, Fig. 1E). Mean CL, the volume of serum antibody cleared per unit of time, was approximately 1.2-fold lower for the YTE variant compared with WT in mice (WT, CL = 0.01063 ± 0.0029 mL/min/kg; YTE, CL = 0.00897 ± 0.00224 mL/min/kg; *P* < 0.05; Table 1, Fig. 1E), indicating a significant decrease in the clearance of the YTE variant. Since the area under the curve (AUC) is inversely proportional to CL, the area under the concentration-time curve extrapolated from time zero to infinity (AUC_inf_) was ~1.2-fold higher for the YTE variant (20,100 ± 6,730 h·μg/mL) than for WT huE6F6 (17,200 ± 6,240 h·μg/mL, *P* < 0.05, Table 1, Fig. 1E), indicating a significant increase in the total exposure of the YTE variant in mice.

**Table 1:** Pharmacokinetic parameters of WT huE6F6 and YTE Fc variant in mice and cynomolgus monkeys, calculated using non-compartmental analysis model 200-202 of Phoenix WinNonlin version 6.3

Parameter	Mice		Cynomolgus monkeys			
	WT^a^ * n* = 6	YTE^a^ * n* = 6	WT^b^ * n* = 3	YTE^b^ * n* = 3	WT^c^ * n* = 3	YTE^c^ * n* = 3
CL (mL/min/kg)	0.01063 ± 0.0029	0.00897* ± 0.00224	0.00524 ± 0.00135	0.00247* ± 0.000255	0.00652 ± 0.000504	0.00332* ± 0.00106
AUC_inf_ (h·μg/mL)	17,200 ± 6240	20,100* ± 6730	67,094 ± 20116	136,044* ± 14461	58,342 ± 3816	107,003* ± 32317
t_1/2_ (h)	20.1 ± 7.5	30.9* ± 10.8	126 ± 47	311* ± 14.3	152 ± 32.7	227 ± 140

^a^ Following a single i.v dose of Abs at 10 mg/kg to hFcRn transgenic mice

^b^ Following a single i.v dose of Abs at 20 mg/kg to male cynomolgus monkeys

^c^ Following a single i.v dose of CHO-HBsAg at 3 mg/kg followed by Abs at 20 mg/kg to cynomolgus monkeys

* Indicates a significant increase in AUC_inf_ or t_1/2_ and decrease in CL (*P* < 0.05) of the YTE mutant group compared with WT group

CL, serum clearance; AUC_inf_, area under the concentration-time curve extrapolated from time zero to infinity; t_1/2_, terminal half-life

The group mean ± SD are reported for each parameter

WT huE6F6 and YTE variant were further tested in cynomolgus monkeys (*n* = 3 animals per antibody). Following a single i.v. dose of 20 mg/kg antibody, the PK profile of the YTE variant was found to be distinct from that of the WT. YTE variant exhibited 2.0-fold increased AUC_inf_ (136,044 ± 14,461 h·μg/mL, *P* < 0.05), 2.5-fold prolonged t_1/2_ (311 ± 14.3 h, *P* < 0.05) and 2.1-fold reduced serum clearance (0.00247 ± 0.000255 mL/min/kg, *P* < 0.05) when compared with WT huE6F6 in cynomolgus monkeys (AUC_inf_ = 67,094 ± 20,116 h·μg/mL, t_1/2_ = 126 ± 47 h, CL = 0.00524 ± 0.00135 mL/min/kg) (Table 1, Fig. 1F). When treating with antibody, CHO-HBsAg at 3 mg/kg were implanted into cynomolgus monkeys followed by antibody infusion of WT huE6F6 or YTE variant at 20 mg/kg (*n* = 3 animals per antibody). A remarkable increase in AUC_inf_, 1.8-fold (AUC_inf_ = 107,003 ± 32,317 h·μg/mL, *P* < 0.05) and a significant, 2-fold, decrease in CL (CL = 0.00332 ± 0.00106 mL/min/kg, *P* < 0.05) was observed for the YTE variant as compared to WT (AUC_inf_ = 58,342 ± 3,816 h·μg/mL, CL = 0.00652 ± 0.000504 mL/min/kg) (Table 1, Fig. 1G). Though the YTE variant showed approximately 1.5 times longer terminal half-life than WT (WT, t_1/2_ = 152 ± 32.7 h; YTE, t_1/2_ = 227 ± 140 h) (Fig. 1G), this modest increase was not statistically significant (*P* > 0.05; Table 1, Fig. 1G). As shown in Figure 1H, half-life of CHO-HBsAg in cynomolgus monkeys was appreciably extended (nearly 4-fold compared with parental antibody treatment) following a single i.v dose of CHO-HBsAg at 3 mg/kg followed by a 20 mg/kg dose of YTE variant. This result indicates that the binding of antibody to antigen can prolong the *in vivo* persistence of antigen.

Antibody immunotherapy is a common therapeutic strategy for cancer, patients often receive a single, low intravenous (i.v.) dose of antibody (<10 mg/kg, <600 mg/dose). But for chronic viral infection, the antibody infusion requires a high-level dose (20–30 mg/kg, > 1 g/dose) and more frequent dosing to effectively eradicate the circulating virus, which may induce severe adverse events in patients. Therefore, engineering of huE6F6 to increase its serum half-life offers the potential benefits of greater efficacy, reduce cost, lower dosage and less frequent dosing. It is of great significance for the development of Fc-engineered E6F6-based therapeutics used in CHB treatment.

Introduction of the triple mutation M252Y/S254T/T256E (YTE) into the Fc portion of humanized anti-VEGF antibody (Zalevsky et al., 2010) and anti-RSV antibody (Dall’Acqua et al., 2006) was previously reported to result in a 3.5-fold and 2.5-fold increase, respectively, in the serum half-life in cynomolgus monkeys. The enhancement of half-life (2.5-fold) in cynomolgus monkeys determined for the YTE variant of humanized anti-HBV antibody here is similar to that measured for the same triple mutation in a different humanized IgG1 background (Dall’Acqua et al., 2006). The binding of CHO-HBsAg to antibody results in a significant increase in the plasma antigen concentration, which is due to the recycling or transcytosis of the antibody-antigen complexes by FcRn through the endosomal pathway in cells (Fig. 1H). In contrast, the YTE variant of CHO-HBsAg treatment group induced only a modest, statistically insignificant increase in serum antibody half-life (1.5-fold, *P* > 0.05) (Fig. 1G). We propose that huE6F6-YTE might achieve a maximal increase in serum persistence when targeting HBsAg antigen in cynomolgus monkeys. This limitation may be overcome by a sweeping antibody construct that has both pH-dependent antigen binding and increased binding to cell surface neonatal Fc receptor. Sweeping antibodies are capable of actively eliminating soluble antigens from circulation, and thereby enhance the antibody serum persistence and potentiate *in vivo* efficacy. There are different technologies for generating such antibody including histidine mutagenesis, direct selection from histidine-rich library, and direct identification (Igawa et al., 2016).

This is the first preclinical study to evaluate the pharmacokinetics of an anti-HBV humanized and Fc-modified monoclonal antibody in mice and nonhuman primates, demonstrating a significant increase in serum half-life of up to 300 h with the Fc YTE triple mutation in cynomolgus monkeys. Our *in vivo* pharmacokinetic study has important implications for IgG variants with long half-lives in the CHB clinical setting. Though the pharmacokinetics, safety, and efficacy of this molecule have yet to be studied in humans, our work so far demonstrates its potential benefits for improving compliance with more convenient, long-term dosing. This may ultimately improve the clinical outcome of treatment.

## Electronic supplementary material

Below is the link to the electronic supplementary material.

Supplementary material 1 (PDF 344 kb)
