# Supplementary material for: A novel therapeutic anti-HBV antibody with increased binding to human FcRn improves in vivo PK in mice and monkeys
Source: Protein Cell. 2017 Jul 4;9(1):130–4. doi: 10.1007/s13238-017-0438-y (PMC5777975; doi:10.1007/s13238-017-0438-y)
Supplement: Supplementary file 1 — Supplementary material 1 (PDF 344 kb) [file 13238_2017_438_MOESM1_ESM.pdf]

Materials and Methods

IgG mutagenesis, expression, and purification

Anti-HBsAg humanized E6F6 was used as parental antibody in this study. Five Fc mutants of huE6F6 were produced by site-directed mutagenesis in the C_H2 and C_H3 domains of the Fc region (Figure 1A) followed by incorporation into an IgG1 expression vector. Plasmids containing the variant heavy chain and the wild-type light chain were co-transfected into HEK293E cells at a 1:1 ratio using polyethylenimine (PEI; Polyscience, Chicago, IL) and cultured for 7 days. IgG1 was purified from culture supernatants by protein A affinity chromatography (General Electric Company, Pittsburgh, PA). Protein stability of monomeric IgG1 variants was measured by SDS-PAGE and differential scanning fluorimetry (DSF) using a LightCycler RT-PCR instrument (Roche, South San Francisco, CA).

Chemiluminescent enzyme immunoassay (CLEIA) of HBsAg and hFcRn binding

Intact HBsAg proteins (2 μg/mL) were coated onto 96-well microtiter plates (Corning, Glendale, AZ) in PBS. WT huE6F6 and five Fc variants were initially diluted at 1 μg/mL followed by serial dilution (1:3, 10000.0 – 0.05 ng/mL) across a polypropylene 96-well plate in PBS.

Serial dilution of purified WT huE6F6 and five Fc variants (100 - 0.045 μg/mL) were plated on hFcRn-coated (1 μg/mL) plates in PBS (pH 6.0), and incubated for 1 h at room temperature, prior to washing using PBST (pH 6.0). The chemiluminescent signal was measured to detect bound receptor labeled by horseradish peroxidase (HRP)-conjugated anti-human IgA+IgG+IgM (H+L) antibody (KPL, Gaithersburg, MD). The same protocol was performed at pH 7.4. EC_{50} titers, representing the concentration for 50% of maximal binding, were calculated by GraphPad Prism (GraphPad Software, San Diego, CA).

.

hFcRn construct and confocal microscopy

To produce the hFcRn stable construct, the sequences encoding the N-terminal EGFP-tagged hFcRn construct comprising signal sequence, distinct α-chains (FCGRT; Uniprot: P55899) and β_2-microglobulin (B2MG; Uniprot: P61769) were PCR-amplified from corresponding cDNA (kindly provided by Han Lab of Xiamen University). This PCR product was digested with *Bam*HI and *Xho*I, and inserted between the restriction sites of pBoBi CS 2.0 lentivirus vector (also kindly provided by Han Lab of Xiamen University). Lentiviral constructs were co-transfected into MDCK epithelial cells with three other packaging plasmids: pMDL, VSV-G, and Rev at a ratio of 5:5:3:2. The MDCK cell line was then screened by flow-cytometry and validated using PE labeled anti-human β_2-microglobulin antibody (Biolegend, San Diego, CA). Dylight-594 labeled human IgG (Pierce, Waltham, MA) was used to examine the pattern of hIgG binding with hFcRn. All expression patterns were determined using immunofluorescence (IF) imaging, which was performed by standard protocols using an InCell Analyzer (GE, Fairfield, CT) in confocal mode. To assess pH dependence of antibody binding to hFcRn by flow cytometry, MDCK cells were incubated and stained with 1 μg Dylight-594 labeled human IgG at pH 6.0 and pH 7.4.

Competitive binding assay

WT huE6F6 and Fc mutants were tested in a flow cytometry-based competitive binding assay through competition with 1 μg Dylight-594 labeled human IgG. Briefly, 2×10^5 hFcRn-expressing MDCK cells were washed twice with FACS binding buffer (PBS containing 1% bovine serum albumin, pH 7.4), then re-suspended in 100 μL titrated aliquots of WT huE6F6 or Fc mutants (3-fold serial dilutions from 200 to 0.27 μg/mL) and concentrated supernatant containing 1 μg Dylight-594 labeled human IgG in FACS binding buffer, pH 6.0. After 1h incubation on ice, the cells were washed twice again with FACS binding buffer, pH 6.0, and analyzed by flow cytometry using a Facsaria III flow cytometer (BD Biosciences, San Jose, CA). IC_{50} values were calculated using GraphPad Prism (GraphPad Software, San Diego, CA).

Pharmacokinetic study in mice

Human FcRn transgenic mice used in this study are knockout mice carrying hFcRn cDNA transgenic line designated C57BL/6-Fcgrt$^{tm1(hFCGRT)}$/Bcgen (Biocytogen, Beijing, China). All mice were maintained under specific pathogen-free conditions in the Laboratory Animal Centre of Xiamen University. The experiments were conducted in accordance with the *Guide for the Care and Use of Laboratory Animals*.

We compared *in vivo* pharmacokinetics of WT huE6F6 and huE6F6-YTE in both mice and cynomolgus monkeys. Sex-matched (6~8-week-old) hFcRn transgenic mice were given an i.v. bolus dose of 10 mg/kg antibody on day 0. Six mice were used for per antibody. Blood samples were obtained from the retro-orbital plexus using capillary pipettes at different time points throughout the 2-week studies. A quantitative CLEIA assay of HBsAg specific binding as described above was used to monitor the serum concentrations of the tested antibodies.

Pharmacokinetic study in cynomolgus monkeys

The PK study in cynomolgus monkeys was approved by the Institutional Animal Care and Use Committee of Xiamen University, and conducted at WuXi AppTec.,Ltd (WuXi AppTec, Wuxi, Jiangsu). Three cynomolgus monkeys were assigned to each of the two antibody groups and given a single, 20 mg/kg i.v. injection of WT huE6F6 or huE6F6-YTE. For the antibody treatment group, three cynomolgus monkeys were assigned to each of the two antibody groups and given a single i.v. dose of CHO-HBsAg at 3 mg/kg followed by antibody treatment of WT huE6F6 or huE6F6-YTE at 20 mg/kg. Blood samples were drawn prior to dosing on day 0; at 0.083, 0.5, 1, 2, 4, 10 and 24 h after dosing; and at 48, 72, 96, 144, 192, 240, 336, 408, 504 and 672 h after dosing. The serum concentrations of antibodies were determined using HBsAg specific binding CLEIA as described above, with a separate standard curve generated for each variant test.

Pharmacokinetics analysis

Data collected from the PK studies of hFcRn transgenic mice and the cynomolgus monkeys were analyzed using non-compartmental analysis model 200 - 202 of Phoenix WinNonlin version 6.3 (Certara, Princeton, NJ). AUC_{inf} (area under the curve to infinity) was calculated using the log-linear trapezoidal method. Terminal half-life ($t_{1/2}$) was calculated using log-linear regression of the concentration data, including at least the last three sampling time points with measurable concentrations. Serum clearance was estimated as CL = dose/AUC_{inf}. Descriptive statistics for major PK parameters were then calculated.

Statistical Analysis

Statistical comparison between different groups was performed by the Student t test using GraphPad Prism. A P value $<.05$ was considered statistically significant.
